# Burden and Outcomes of Acute Kidney Injury in Critically Ill Patients

**DOI:** 10.7759/cureus.110291

**Published:** 2026-06-05

**Authors:** Anakath M Ijas, Gayathry Rajasekharan, Chinthu Sara Jacob, Vijayakumar Anil, Rajasekharan Chandrasekharan

**Affiliations:** 1 Internal Medicine, Malabar Medical College Hospital and Research Centre, Kozhikode, IND; 2 Pathology, Government Medical College, Thrissur, Thrissur, IND; 3 Internal Medicine, Sree Uthradom Thirunal (SUT) Academy of Medical Sciences, Trivandrum, IND; 4 Internal Medicine, Azeezia Institute of Medical Sciences & Research, Kollam, IND; 5 General Medicine, Sree Uthradom Thirunal (SUT) Academy of Medical Sciences, Trivandrum, IND

**Keywords:** acute kidney injury, community-acquired acute kidney injury (caaki), hospital-acquired acute kidney injury (haaki), intensive care unit, kdigo criteria, renal replacement therapy, sepsis

## Abstract

Introduction

Acute kidney injury (AKI) represents a frequent and serious complication among patients requiring intensive care. This syndrome is a primary driver of elevated morbidity and mortality, while also leading to extended hospital stays and an increased susceptibility to future chronic kidney disease (CKD). Early identification of both risk factors and etiological factors is essential for timely intervention and improved clinical outcomes. The study aimed to evaluate the clinical characteristics, demographic profiles, and etiological risk factors of critically ill patients presenting with AKI and compare the outcomes of patients with and without sepsis.

Methods

A longitudinal observational study was conducted in the medical and surgical intensive care units (ICUs) of a tertiary care hospital in Kerala between February 2021 and August 2022. A total of 77 adult patients diagnosed with AKI based on the Kidney Disease: Improving Global Outcomes (KDIGO) criteria were included in the study. Clinical characteristics, laboratory parameters, etiologies, management strategies, and outcomes were analyzed using IBM SPSS Statistics software, version 25 (IBM Corp., Armonk, NY, USA). A p-value of less than 0.05 was considered statistically significant.

Results

The overall mortality rate within the enrolled AKI cohort was 21 (27.3%). The majority of the patients were male (50, 64.9%) and over the age of 60 years (35, 45.4%). Community-acquired AKI (CAAKI) constituted 56 (72.7%) of the cases, while hospital-acquired AKI (HAAKI) accounted for 21 (27.2%) cases. Sepsis was identified as the leading cause of AKI at 26 (33.7%), followed by poisoning (13, 16.9%) and cardiac etiologies (14, 18.2%). Mortality was not significantly associated with age, gender, AKI type, or laboratory parameters, including hemoglobin levels or thrombocytopenia. However, mortality was significantly associated with metabolic acidosis (p=0.004), hyperbilirubinemia (p=0.037), and higher KDIGO stages (chi-square (χ²)=6.11, p=0.047). Among the 56 survivors, complete renal recovery was achieved in 47 patients (84.8%), partial recovery was observed in six patients (10.4%), and three patients (5.4%) remained dialysis-dependent at the follow-up period.

Conclusion

AKI remains a pervasive complication among the critically ill, where it continues to be linked with considerable fatality rates within the intensive care setting. Sepsis is the predominant etiology. Higher KDIGO stages, metabolic acidosis, and hyperbilirubinemia are significant predictors of poor outcomes. Early detection and prompt management of reversible factors may significantly improve survival rates.

## Introduction

Acute kidney injury (AKI) represents a common and serious clinical condition marked by an abrupt decline in renal performance. This failure leads to the systemic retention of nitrogenous waste, electrolyte instability, and acid-base derangements. Clinically, AKI is diagnosed through rising serum creatinine concentrations, diminished urinary output, or both. Within the intensive care unit (ICU), AKI occurs frequently and serves as a major driver of patient morbidity and lethal outcomes [1]. Even mild forms of AKI have been linked to an increased risk of mortality, emphasizing its clinical relevance [2].

Globally, AKI affects 30%-50% of ICU admissions, prolongs hospitalization, and increases healthcare costs [2]. It is correlated with prolonged hospitalization, increased healthcare costs, and unfavorable clinical outcomes. In addition to acute injury, patients often face long-term health challenges; AKI is strongly linked to the subsequent development of chronic renal impairment, permanent kidney failure, and heightened cardiovascular vulnerability [3-4].

The epidemiology of AKI varies across regions due to differences in healthcare infrastructure, socioeconomic factors, and disease patterns. In India, particularly Kerala, infections, toxins, and tropical illnesses remain important causes, while hospital-acquired AKI (HAAKI) is often linked to sepsis, surgery, and nephrotoxic drugs [5]. AKI can be classified as either community-acquired AKI (CAAKI) or HAAKI, each with distinct etiological profiles. Among critically ill patients, sepsis remains the leading cause, with a complex pathophysiology that involves inflammation, endothelial dysfunction, and microcirculatory alterations [6].

The Kidney Disease: Improving Global Outcomes (KDIGO) criteria have standardized AKI diagnosis and staging, yet challenges persist due to delayed biomarker elevation and variability in application across healthcare settings [7]. However, the challenge of early diagnosis persists, primarily due to the delayed elevation of conventional biomarkers such as serum creatinine. Critical AKI episodes, especially those requiring renal replacement therapy (RRT), are associated with mortality rates between 40% and 60%. Furthermore, a significant proportion of surviving patients do not regain their pre-morbid kidney function [2].

With strong healthcare indicators and a high burden of non-communicable diseases, Kerala serves as a suitable setting for studying AKI in critically ill patients. [8]. However, there is a notable scarcity of comprehensive region-specific data on the incidence, etiology, and outcomes of AKI.

In spite of the progress made in understanding AKI, several gaps remain, particularly in the context of critically ill populations in India and specifically in Kerala.

Most of the current research is retrospective or cross-sectional, predominantly concentrating on short-term outcomes such as in-hospital mortality. Such study designs do not adequately capture the longitudinal course of AKI, which includes aspects like renal recovery, progression to chronic kidney disease (CKD), recurrent episodes, and long-term mortality. Thus, there is a dearth of prospective longitudinal studies addressing these critical outcomes [3].

Although the KDIGO criteria have improved standardization, the variability in their application across healthcare settings leads to inconsistencies in reported incidence and outcomes [9]. Variations in monitoring practices and definitions limit comparability across studies and highlight the need for uniform methodologies. Region-specific evidence from Kerala remains scarce despite its unique epidemiological profile. [10].

Moreover, AKI in critically ill patients is often multifactorial; however, numerous studies focus on isolated causes such as sepsis, neglecting the evaluation of interactions with comorbidities, drug exposures, and hemodynamic factors. There is an urgent need for comprehensive analyses that integrate these variables to better identify outcome predictors.

Finally, limited attention has been given to post-discharge outcomes and follow-up care. AKI survivors continue to face an increased risk of developing CKD and cardiovascular complications; however, structured long-term management remains inadequate [4].

The objective of this study was to evaluate the clinical characteristics, demographic profiles, and etiological risk factors of critically ill patients presenting with AKI. Furthermore, the study aimed to compare the proportion of patients with and without sepsis developing AKI.

## Materials and methods

This longitudinal observational study was conducted in the medical and surgical ICUs of Azeezia Medical Institute of Medical Sciences and Research, located in Kollam, Kerala. The study, conducted from February 2021 to August 2022, was designed to determine the proportion of critically ill patients who develop AKI, identify its associated etiological risk factors, and compare the outcomes of patients with and without sepsis.

The study population consisted of adult patients with AKI admitted to various medical and surgical ICUs at Azeezia Medical Institute of Medical Sciences and Research. This included all adult ICU patients who were actively monitored and diagnosed with AKI within the first seven days of their ICU hospitalization, based on the KDIGO criteria. Consequently, a total of 77 patients fulfilling the KDIGO criteria for AKI were enrolled using purposive sampling [7].

The inclusion criteria encompassed adult ICU patients who provided informed consent and met the standardized KDIGO diagnostic criteria for AKI (based on elevations in serum creatinine, reduction in urine output, or both). Clinical features such as oliguria, anuria, or uremic symptoms were noted during history taking but were not used as restrictive filters to exclude non-oliguric AKI [7].

The exclusion criteria included patients with pre-existing CKD, bilaterally small kidneys observed on ultrasonography, and chronic obstructive nephropathy.

A comprehensive clinical history, including a physical examination, laboratory parameters, and imaging findings, was recorded. Serum creatinine levels and urine output were measured, and an AKI diagnosis was made according to KDIGO guidelines. A sepsis workup, including a complete blood count, C-reactive protein (CRP), procalcitonin, and serum sodium, potassium, calcium, phosphorus, and uric acid, was performed. Arterial blood gas estimation and urine routine were also performed. Two blood culture samples were taken from different sites simultaneously. Additionally, a tropical fever workup, including dengue IgM, *Leptospira* IgM, scrub typhus IgM, and malaria parasites, was conducted. Patient progress was assessed based on serial measurements of serum creatinine and urine output. On the seventh day and at the one-month follow-up, patients were categorized based on their stage of recovery as completely recovered, partially recovered, or dialysis-dependent.

Statistical analysis was performed using IBM SPSS Statistics software, version 25 (IBM Corp., Armonk, NY, USA). Continuous variables were expressed as mean ± standard deviation (SD), while categorical variables were presented as frequencies and percentages. The chi-square test (χ²) was employed to analyze the associations between categorical clinical characteristics, laboratory parameters, staging, interventions, and mortality outcomes. A p-value < 0.05 was considered statistically significant.

Clearance was obtained from the Institutional Ethics Committee of Azeezia Institute of Medical Sciences & Research (AEC/REV/2021/35). Because this was a non-interventional, longitudinal observational study, written informed consent was obtained from the patients or their relatives (if the patient was in an altered sensorium/comatose) prior to data collection. An ethics waiver was not requested or required. The confidentiality and anonymity of patient information were maintained during and after the study. 

## Results

A total of 77 critically ill patients diagnosed with AKI were included. The majority of these patients were male (50, 64.9%), and a significant proportion were aged over 60 years (35, 45.4%). However, no statistically significant association was found between gender (χ²=0.001, p=0.965) or age group and mortality (χ²=0.312, p=0.989). The gender and age distribution of both survivors and non-survivors is summarized in Table 1.

**Table 1:** Gender and Age Distribution of Survivors and Non-survivors Demographic variables are presented as N (%) representing the frequency and percentage within each group. Percentages were calculated column-wise using survivor and non-survivor totals. P-values were derived using Pearson’s chi-square (χ^2^) test to compare the distribution of gender and age groups between survivors and non-survivors.

Characteristic	Survivors N (%)	Non-survivors N (%)	Total N (%)	Test Statistic	p-value
Total Patients	56 (72.7%)	21 (27.3%)	77 (100%)	-	-
Gender	-	-	-	χ^2^=0.001	0.965
Male	37 (66.1%)	13 (61.9%)	50 (64.9%)	-	-
Female	19 (33.9%)	8 (38.1%)	27 (35.1%)	-	-
Age Group, in Years	-	-	-	χ^2^=0.312	0.989
18–30	4 (7.14%)	1 (4.8%)	5 (6.5%)	-	-
31–40	5 (8.9%)	4 (19.0%)	9 (11.7%)	-	-
41–50	9 (16.1%)	4 (19.0%)	13 (16.9%)	-	-
51–60	12 (21.4%)	3 (14.3%)	15 (19.5%)	-	-
> 60	26 (46.4%)	9 (42.9%)	35 (45.4%)	-	-

Clinical and etiological distribution

Most patients were admitted to the medical ICU (MICU), accounting for 57 (74.0%), with fewer in the surgical ICU (SICU), 20, 25.9%). CAAKI was found to be predominant, accounting for 56 (72.7%) patients, while HAAKI accounted for 21 (27.3%) patients, with no significant difference in mortality observed based on ICU type (p=0.188) or AKI type (p=0.102).

Sepsis was the leading etiology at 26 (33.7%), followed by cardiac causes at 14 (18.2%), poisoning/toxins at 13 (16.9%), drug-induced causes at six (7.8%), and tropical infections at five (6.5%). While cardiac causes showed a higher proportion of non-survivors at nine (42.9%), the overall distribution of etiologies did not show a statistically significant association with mortality (p>0.05). The subcategories of sepsis included cellulitis, urosepsis, pneumonia, peritonitis, cholangitis, septic arthritis, and spontaneous bacterial peritonitis. The breakdown of ICU admission types, AKI classifications, and primary etiologies is presented in Table 2.

**Table 2:** ICU Admission, Type of AKI, and Etiological Distribution Among Survivors and Non-survivors Categorical variables are presented as N (%) representing the frequency and percentage within each group. P-values were derived using Pearson’s chi-square (χ2) test to assess the association between clinical characteristics and mortality status. A p-value of <0.05 is considered statistically significant. MICU: medical ICU; SICU: surgical ICU; AKI: acute kidney injury; CAAKI: community-acquired AKI; HAAKI: hospital-acquired AKI

Characteristic	Survivors N (%)	Non-survivors N (%)	Total N (%)	Test statistic	p-value
ICU Type	-	-	-	χ^2=^1.73	0.188
MICU	42 (75.0%)	15 (71.4%)	57 (74.0%)	-	-
SICU	14 (25.0%)	6 (28.5%)	20 (25.9%)	-	-
Type of AKI	-	-	-	χ^2=^2.67	0.102
CAAKI	40 (71.4%)	16 (76.1%)	56 (72.7%)	-	-
HAAKI	16 (28.6%)	5 (23.8%)	21 (27.3%)	-	-
Etiology of AKI	-	-	-	χ^2^=0.579	>0.05
Sepsis	19 (33.9%)	7 (33.3%)	26 (33.8%)	-	-
Poisoning/Toxins	9 (16.07%)	4 (19.0%)	13 (16.9%)	-	-
Cardiac Causes	5 (8.9%)	9 (42.9%)	14 (18.2%)	-	-
Drug-Induced	5 (8.9%)	1 (4.7%)	6 (7.8%)	-	-
Tropical Infections	4 (7.14%)	1 (4.7%)	5 (6.5%)	-	-

Laboratory predictors of mortality

Several laboratory variables were associated with clinical outcomes. Survivors were more likely to have hemoglobin > 10 g/dl (n=40, 71.4%) and platelet counts > 1.5 lakh/mm³ (n=34, 60.7%), while non-survivors more frequently had hemoglobin < 10 g/dl (n=9, 42.9%) and thrombocytopenia (n=11, 52.3%), but these associations did not reach statistical significance (p>0.05).

Metabolic acidosis was present in 19 (90.4%) non-survivors compared to 29 (51.7%) survivors, representing a statistically significant association (χ²=8.15, p=0.004). Hyperbilirubinemia (> 2 mg/dl) was observed in 10 (47.6%) non-survivors compared to 13 (23.2%) survivors; the association reached statistical significance (p=0.037), as explained in Table 3.

**Table 3:** Laboratory Predictors of Mortality Among Survivors and Non-survivors Categorical variables are presented as N (%) representing the frequency and percentage within each group. P-values were derived using Pearson’s chi-square (χ^2^) test. A p-value of <0.05 is considered statistically significant.

Laboratory Parameter	Survivors N (%)	Non-survivors N (%)	Total N (%)	Test statistic	p-value
Hemoglobin	-	-	-	χ^2^=0.811	0.368
> 10g/dl	40 (71.4%)	12 (57.1%)	52 (67.5%)	-	-
< 10g/dl	16 (28.5%)	9 (42.8%)	25 (32.4%)	-	-
Platelet Count	-	-		χ^2^=0.0647	0.799
> 1.5 lakh/mm^3^	34 (60.7%)	10 (47.6%)	44 (57.1%)	-	-
< 1.5 lakh/mm^3^	22 (39.2%)	11 (52.3%)	33 (42.8%)	-	-
Metabolic Acidosis	-	-		χ^2^=8.15	0.004
Present	29 (51.7%)	19 (90.4%)	48(62.3%)	-	-
Absent	27 (48%)	2 (9.5%)	29(37.6%)	-	-
Hyperbilirubinemia	-	-		χ^2=^4.34	0.037
Normal	43 (76.7%)	11 (52.3%)	54 (70.1%)	-	-
> 2 mg/dl	13 (23.2%)	10 (47.6%)	23 (29.8%)	-	-

Electrolyte abnormalities

Hyponatremia, hyperkalemia, and hypokalemia were frequently observed within the cohort, with potassium imbalances showing a statistically significant variation between survivors and non-survivors (p<0.001).

Interventions and severity

The severity of illness was reflected in the level of supportive therapy required and the KDIGO staging. Inotrope use was required for 16 (76.1%) non-survivors compared to 33 (58.9%) survivors, though this difference did not reach statistical significance (p=0.163). Dialysis was required for 11 (52.3%) non-survivors compared to 17 (30.3%) survivors (χ²=3.196, p=0.074).

Higher KDIGO stages were strongly associated with mortality (χ²=6.11, p=0.047), with Stage 3 accounting for 16 (76.1%) non-survivors compared to 25 (44.6%) survivors.

Mortality rates escalated progressively with increasing KDIGO stages. Stage 1 accounted for two (9.5%) deaths, Stage 2 for 3 (14.2%), and Stage 3 for 16 (76.1%). Among the 56 survivors, complete renal recovery was achieved in 47 patients (84.8%), partial recovery was observed in six patients (10.4%), and three patients (5.4%) remained dialysis dependent at the follow-up period. The correlation between disease stage, dialysis, inotrope use, and patient outcomes is detailed in Table 4.

**Table 4:** Supportive Therapy and AKI Staging in Relation to Mortality Percentages are calculated based on the total number of survivors (n = 56) and non-survivors (n = 21). P-values were derived using Pearson’s chi-square (χ^2^) test. A p-value of <0.05 is considered statistically significant. KDIGO: Kidney Disease: Improving Global Outcomes; AKI: acute kidney injury

Intervention	Survivors; Yes, N (%)	Non-survivors, Yes, N (%)	Total N (%)	Test Statistic	p-value
Inotropes	33 (58.9%)	16 (76.1%)	49(63.6%)	χ^2=^1.94	0.163
Dialysis	17 (30.3%)	11 (52.3%)	28(36.3%)	χ^2=^3.196	0.074
KDIGO Stage	-	-	-	χ^2=^6.11	0.047
Stage 1	13 (23.2%)	2 (9.5%)	15 (19.4%)	-	-
Stage 2	18 (32.1%)	3 (14.2%)	21 (27.2%)	-	-
Stage 3	25 (44.6%)	16 (76.1%)	41 (53.2%)	-	-

## Discussion

AKI continues to be a significant factor contributing to morbidity and mortality among critically ill patients worldwide. This longitudinal observational study offers valuable insights into the clinical characteristics, etiological profiles, and outcome predictors of AKI in an ICU setting in Kerala. Despite advancements in critical care, the findings reaffirm that AKI remains linked to substantial mortality and adverse clinical outcomes [11].

The overall mortality rate within the enrolled AKI cohort was 21 (27.3%), which aligns with findings from previous ICU-based studies conducted in India and other developing regions, where mortality rates range between 25% and 40% depending on the illness severity and underlying etiology [12-13]. This underscores that AKI in critically ill patients is frequently part of a broader syndrome of multiorgan dysfunction, with mortality rates influenced by the severity of systemic illness, hemodynamic instability, and delays in intervention.

A predominance of males (50, 64.9%) was noted in this cohort, which corresponds with earlier epidemiological studies [14-15]. This may be attributed to increased exposure to environmental and occupational risk factors, as well as a greater prevalence of comorbidities such as hypertension and cardiovascular disease among males. Additionally, sociocultural differences related to healthcare access and utilization may contribute to this pattern.

The majority of patients in the present study were elderly (> 60 years) at 35 (45.4%), highlighting age as a common baseline distributional characteristic within the ICU AKI population. However, age did not act as a statistically significant predictor of mortality in this cohort. These demographic findings are summarized in Table 1. The age-related decline in renal functional reserve, reduced renal perfusion, and the increased burden of comorbidities such as diabetes and cardiovascular disease make this demographic more susceptible to renal injury [16]. Previous research has demonstrated that the incidence of AKI in elderly septic patients may exceed 50%, with worse outcomes compared to younger individuals [17].

A notable finding of this study is the predominance of CAAKI (56, 72.7%) compared to HAAKI. This finding reflects the persistent challenge posed by preventable factors leading to AKI in developing regions, such as infections, dehydration, and toxin exposure. Data from international registries, including those from the International Society of Nephrology, have similarly highlighted the high prevalence of CAAKI in South Asia [18]. These results emphasize the need for strengthening primary healthcare systems and early referral mechanisms.

In this study, sepsis was identified as the primary etiology of AKI present in 26 (33.7%) of cases. These findings are summarized in Table 2. This finding aligns with international literature, which suggests that sepsis is responsible for nearly 40% to 50% of AKI instances within intensive care units [19-20]. The pathophysiology of sepsis-associated AKI is complex, involving systemic inflammation, endothelial dysfunction, microvascular alterations, and mitochondrial injury. Furthermore, the bidirectional relationship between sepsis and AKI contributes to a vicious cycle of worsening organ dysfunction and increased mortality rates [21-22]. Therefore, early recognition and prompt management of sepsis remain crucial in mitigating AKI-related mortality.

Toxin-induced AKI accounted for 13 (16.9%) of cases, reflecting regional epidemiological patterns. The exposure to agricultural chemicals, snake envenomation, and various environmental toxins continues to be a significant contributor to AKI in tropical regions [23]. The mechanisms through which toxins induce renal injury include direct nephrotoxicity, hemolysis, rhabdomyolysis, and disseminated intravascular coagulation, highlighting the need for targeted preventive strategies.

Cardiac causes contributed to 14 (18.2%) of AKI cases, underscoring the importance of cardiorenal interactions in critically ill patients. Reduced cardiac output and renal hypoperfusion in conditions such as congestive heart failure can trigger AKI, a phenomenon described as cardiorenal syndrome [24]. The coexistence of cardiac and renal dysfunction is associated with increased morbidity and mortality.

Laboratory abnormalities were significant predictors of outcomes in this study. Anemia and thrombocytopenia were more prevalent among non-survivors, as mentioned in Table 3, likely indicative of systemic inflammation, sepsis, or coagulopathy. Additionally, electrolyte disturbances involving potassium levels varied significantly between outcomes (p<0.001), a finding critical to monitor due to the known capacity of severe imbalances to induce life-threatening cardiac arrhythmias [25].

Metabolic acidosis emerged as a strong predictor of mortality, observed in the majority of non-survivors. This finding is clinically relevant, as metabolic acidosis reflects both impaired renal acid excretion and systemic hypoperfusion. Severe acidosis can adversely affect cardiovascular function, reduce myocardial contractility, and impair cellular metabolism, thereby contributing to unfavorable outcomes [26].

Elevated bilirubin levels were also notably correlated with mortality, indicating the presence of hepatic dysfunction or multiorgan failure. The coexistence of renal and hepatic dysfunction is a well-recognized marker of disease severity and is associated with significantly increased mortality in critically ill patients [27].

The necessity for inotropic support and RRT was significantly higher among non-survivors, which is summarized in Table 4, demonstrating the relationship between supportive therapy, KDIGO staging, and mortality rates. These interventions reflect severe hemodynamic instability and advanced renal dysfunction, both of which are known predictors of poor prognosis. Similarly, the KDIGO staging showed a strong correlation with outcomes, with the majority of non-survivors classified as Stage 3. This supports the utility of KDIGO classification as a reliable prognostic tool [28].

Encouragingly, among the 56 survivors, complete renal recovery was achieved in 47 patients (84.8%), partial recovery was observed in six patients (10.4%), and three patients (5.4%) remained dialysis dependent, highlighting the reversible nature of AKI when timely and appropriate management is instituted [29]. This finding reinforces the relevance of early detection, avoidance of nephrotoxic insults, and prompt rectification of underlying causes.

Preventive strategies are imperative for minimizing the incidence and severity of AKI. A considerable number of HAAKI cases have been linked to nephrotoxic medications, emphasizing the need for careful drug monitoring and dosage adjustments in high-risk patients [30]. Ensuring adequate hydration and hemodynamic stability are also essential components of prevention.

Limitations

The study’s primary constraints included a limited sample size of 77 adult patients and a relatively brief duration of 18 months. Conducted within a single tertiary care center in Kerala, the research was geographically restricted, and the follow-up period was limited to merely one month. Additionally, clinical challenges such as the difficulty of identifying precise AKI etiologies and the cautious approach to initiating RRT, often delayed due to concerns over complications like hypotension, may have influenced the management data.

## Conclusions

This study underscores the clinical significance of AKI in critically ill patients and emphasizes the need for early detection and evidence-based management strategies to improve outcomes. Sepsis emerged as the predominant etiological factor, underscoring its central role in the pathogenesis of AKI in intensive care settings. Factors such as advanced age and comorbidities, including diabetes, hypertension, and chronic liver disease, were key predisposing factors. Mortality was strongly predicted by a higher AKI stage as per KDIGO criteria, metabolic acidosis, and hyperbilirubinemia. While the requirement for inotropic support and the need for RRT showed a trend towards higher mortality, they did not reach statistical significance. Additionally, laboratory abnormalities, including anemia, thrombocytopenia, and electrolyte imbalances involving potassium levels, were statistically significant. Notably, a substantial proportion of survivors achieved complete renal recovery, highlighting the potential reversibility of AKI with timely intervention. These results emphasize the importance of early identification of high-risk patients, prompt management of sepsis, and vigilant monitoring to improve clinical outcomes in critically ill populations.

## References

[1] Kellum JA, Romagnani P, Ashuntantang G, Ronco C, Zarbock A, Anders HJ (2021). Acute kidney injury. Nat Rev Dis Primers.

[2] Hoste EA, Bagshaw SM, Bellomo R (2015). Epidemiology of acute kidney injury in critically ill patients: the multinational AKI-EPI study. Intensive Care Med.

[3] Chawla LS, Eggers PW, Star RA, Kimmel PL (2014). Acute kidney injury and chronic kidney disease as interconnected syndromes. N Engl J Med.

[4] Odutayo A, Wong CX, Farkouh M, Altman DG, Hopewell S, Emdin CA, Hunn BH (2017). AKI and long-term risk for cardiovascular events and mortality. J Am Soc Nephrol.

[5] Eswarappa M, Gireesh MS, Ravi V, Kumar D, Dev G (2014). Spectrum of acute kidney injury in critically ill patients: a single center study from South India. Indian J Nephrol.

[6] Bellomo R, Kellum JA, Ronco C (2017). Acute kidney injury in sepsis. Intensive Care Med.

[7] (2012). Notice. Kidney Int Suppl (2011).

[8] Sarma PS, Sadanandan R, Thulaseedharan JV (2019). Prevalence of risk factors of non-communicable diseases in Kerala, India: results of a cross-sectional study. BMJ Open.

[9] Wiersema R, Jukarainen S, Eck RJ (2020). Different applications of the KDIGO criteria for AKI lead to different incidences in critically ill patients: a post hoc analysis from the prospective observational SICS-II study. Crit Care.

[10] India State-Level Disease Burden Initiative Collaborators (2017). Nations within a nation: variations in epidemiological transition across the states of India, 1990-2016 in the Global Burden of Disease Study. Lancet.

[11] Bhattacharjee K, Mukherjee T, Kundu AK (2026). Epidemiology and outcome of acute kidney injury in critical care unit: a prospective observational study [IN PRESS]. Indian J Nephrol.

[12] Korula S, Balakrishnan S, Sundar S, Paul V, Balagopal A (2016). Acute kidney injury-incidence, prognostic factors, and outcome of patients in an intensive care unit in a tertiary center: a prospective observational study. Indian J Crit Care Med.

[13] Havaldar A, Sushmitha EA, Shrouf SB, Monisha HS, Madhammal N, Selvam S (2024). Epidemiological study of hospital acquired acute kidney injury and its effect on the outcome in patients admitted to the ICU (HAAKI). Indian J Crit Care Med.

[14] Priyamvada PS, Jayasurya R, Shankar V, Parameswaran S (2018). Epidemiology and outcomes of acute kidney injury in critically ill: experience from a tertiary care center. Indian J Nephrol.

[15] Tangchitthavorngul S, Lumlertgul N, Peerapornratana S (2025). Epidemiology and long-term outcomes of critically ill patients with severe AKI in India and Southeast Asia. Intensive Care Med.

[16] Prasad G, Deepankar P, Choudhary MK, Ahmad A, Ram B, Kumar N, Patel PS (2024). Clinical profile and short-term outcomes of acute kidney injury in elderly patients in a tertiary care center. Cureus.

[17] Wang J, Li J, Wang Y (2019). [Clinical characteristics and prognosis of acute kidney injury in elderly patients with sepsis]. Zhonghua Wei Zhong Bing Ji Jiu Yi Xue.

[18] Prasad N, Jaiswal A, Meyyappan J (2024). Community-acquired acute kidney injury in India: data from ISN-acute kidney injury registry. Lancet Reg Health Southeast Asia.

[19] White KC, Serpa-Neto A, Hurford R (2023). Sepsis-associated acute kidney injury in the intensive care unit: incidence, patient characteristics, timing, trajectory, treatment, and associated outcomes. A multicenter, observational study. Intensive Care Med.

[20] Takeuchi T, Flannery AH, Liu LJ (2025). Epidemiology of sepsis-associated acute kidney injury in the ICU with contemporary consensus definitions. Crit Care.

[21] Yadla M, Babu NM (2026). Outcomes of sepsis-associated acute kidney injury. Indian J Nephrol.

[22] Shi B, Ye J, Chen W, Liao B, Gu W, Yin H, Lyu J (2025). Prognosis of critically ill patients with early and late sepsis-associated acute kidney injury: an observational study based on the MIMIC-IV. Ren Fail.

[23] Khan A, Quaiser S, Khan R, Agrawal N (2025). Poisoning and envenomation induced acute kidney injury: a hospital-based study. Indian J Nephrol.

[24] Dutta A, Saha S, Bahl A, Mittal A, Basak T (2023). A comprehensive review of acute cardio-renal syndrome: need for novel biomarkers. Front Pharmacol.

[25] Wyatt NE, Gakhokidze L, Bhave G, Arroyo JP (2025). Hyperkalemia in a patient with resolving acute kidney injury. Am J Kidney Dis.

[26] Yagi K, Fujii T (2021). Management of acute metabolic acidosis in the ICU: sodium bicarbonate and renal replacement therapy. Crit Care.

[27] Choi JS, Chung KS, Lee EH (2020). The role of bilirubin to albumin ratio as a predictor for mortality in critically ill patients without existing liver or biliary tract disease. Acute Crit Care.

[28] Khwaja A (2012). KDIGO clinical practice guidelines for acute kidney injury. Nephron Clin Pract.

[29] Ostermann M, Lumlertgul N, Jeong R, See E, Joannidis M, James M (2025). Acute kidney injury. Lancet.

[30] Perazella MA, Rosner MH (2022). Drug-induced acute kidney injury. Clin J Am Soc Nephrol.

